# Combined *Lycium babarum* polysaccharides and C-phycocyanin increase gastric *Bifidobacterium* relative abundance and protect against gastric ulcer caused by aspirin in rats

**DOI:** 10.1186/s12986-020-00538-9

**Published:** 2021-01-06

**Authors:** Shu-Yu Hsieh, Yu Zhi Lian, I-Hsuan Lin, Yu-Chen Yang, Alexey A. Tinkov, Anatoly V. Skalny, Jane C.-J. Chao

**Affiliations:** 1 School of Nutrition and Health Sciences, College of Nutrition, Taipei Medical University, 250 Wu-Hsing Street, Taipei 110, 11031 Taiwan; 2 Research Center of Translational Medicine, Taipei Medical University, Taipei 110, Taiwan; 3 Joint Biobank, Office of Human Research, Taipei Medical University, Taipei 110, Taiwan; 4 Sechenov First Moscow State Medical University, Moscow, Russia; 5 K.G. Razumovsky Moscow State University of Technologies and Management, Moscow, Russia; 6 Master Program in Global Health and Development, Taipei Medical University, Taipei 110, Taiwan; 7 Nutrition Research Center, Taipei Medical University Hospital, Taipei 110, Taiwan

**Keywords:** Aspirin, C-phycocyanin, Gastric ulcer, *Lycium barbarum* polysaccharides, Microbiota

## Abstract

**Background:**

Non-steroidal anti-inflammatory drugs such as aspirin are used for the treatment of cardiovascular disease. Chronic use of low-dose aspirin is associated with the occurrence of gastric ulcer. The aim of this study was to investigate the healing potential of *Lycium barbarum* polysaccharides (LBP) from Chinese Goji berry and C-phycocyanin (CPC) from *Spirulina platensis* on gastric ulcer in rats.

**Methods:**

Male Sprague–Dawley rats were divided into five groups: normal, aspirin (700 mg/kg bw), LBP (aspirin + 100 mg/kg bw/d LBP), CPC (aspirin + 50 mg/kg bw/d CPC), and MIX (aspirin + 50 mg/kg bw/d LBP + 25 mg/kg bw/d CPC) groups. Gastric ulcer was developed by daily oral feeding of aspirin for 8 weeks. Treatments were given orally a week before ulcer induction for 9 weeks.

**Results:**

The MIX group elevated gastric cyclooxygenase-1, prostaglandin E_2_, and total nitrite and nitrate levels by 139%, 86%, and 66%, respectively, compared with the aspirin group (*p* < 0.05). Moreover, the MIX group reduced lipid peroxides malondialdehyde levels by 78% (*p* < 0.05). The treatment of LBP and/or CPC increased gastric *Bifidobacterium* relative abundance by 2.5–4.0 times compared with the aspirin group (*p* < 0.05).

**Conclusions:**

We conclude that combined LBP and CPC enhance gastroprotective factors, inhibit lipid peroxidation, and increase gastric *Bifidobacterium* relative abundance. Combined LBP and CPC have protective potential against gastric ulcer caused by aspirin in rats.

## Introduction

Peptic ulcer is a common disease of the digestive system [1]. Peptic ulcer is characterized by the erosion of mucosa, submucosa, and muscular layers on the lower esophagus, stomach, or duodenum [1]. A systematic study of 31 articles published between January 1980 and February 2009 showed that the pooled incidence rate of uncomplicated peptic ulcer was 0.9 per 1000 person-years in the general population [2]. The lifetime prevalence of peptic ulcer diagnosed by physicians in the western countries was approximately 5–10% in the late twentieth century [3, 4]. The prevalence of peptic ulcer assessed by upper gastrointestinal endoscopy in Taiwan from January to August 2008 was 9.4% [5]. The prevalence of gastric ulcer, duodenal ulcer, as well as both gastric and duodenal ulcers in Taiwan in 2008 was 4.7%, 3.9%, and 0.9%, respectively [5]. Gastric ulcer develops when the gastroprotective factors are overwhelmed by the damaging factors in the stomach [6]. The gastroprotective factors include antioxidants, bicarbonate, mucin, nitric oxide (NO), and prostaglandins (PGs) in the gastric mucosa. The damaging factors include oxidative stress, gastric acid, *Helicobacter pylori*, and non-steroidal anti-inflammatory drugs (NSAIDs). Aspirin (acetylsalicylic acid, PubChem CID: 2244), one of NSAIDs, acts as a deleterious factor. Low-dose (75–325 mg/d) aspirin was used for the protection and therapy of cardiovascular disease. Chronic use of low-dose aspirin was associated with increased ulcer complications [7]. NSAIDs could selectively inhibit gastric cyclooxygenase (COX) activity and decrease gastric PGE_2_ formation [8]. In addition, aspirin could decrease gastric total nitrite and nitrate (NOx) which can act as a protective factor to stimulate gastric blood flow and mucin secretion [9, 10]. On the other hand, aspirin increased oxidative stress by reducing gastric superoxide dismutase activity [11].

*Lycium barbarum* known as Chinese wolfberry or Tibetan goji berry has been used as herbal food for health promotion and traditional Chinese medicine [12]. The water-soluble active components of the extract from the fruit of *Lycium barbarum* L. such as polysaccharides (LBP) have been reported to have antioxidant, anti-inflammatory, and immunomodulatory activity [13–15]. C-phycocyanin (CPC, PubChem SID: 53837743), a major biliprotein pigment of *Spirulina platensis*, was widely used in food coloring and cosmetics as the non-toxic natural dye and has been approved by Food and Drug Administration as a dietary supplement in many countries [16]. C-phycocyanin has been found to possess hepatoprotective, antioxidant, anti-inflammatory, and wound healing properties [16–18]. Due to the characteristics of free oxygen radical scavenger, anti-inflammatory, immunomodulatory, regenerative, and wound-healing activities for LBP and CPC, it is reasonably hypothesized that LBP and/or CPC may exert the recovery of gastric ulcer through lowering oxidative damage, inhibiting pro-inflammatory responses, improving gastroprotection, and altering gastric microbiota [13–20]. The aim of this study was to investigate whether *Lycium barbarum* polysaccharides and/or C-phycocyanin could have healing potential on gastric ulcer caused by aspirin in rats.

## Materials and methods

### Animals and treatments

Male Sprague–Dawley rats (200–250 g) aged 7 weeks old were purchased from BioLASCO Taiwan Co., Ltd. (Taipei, Taiwan), and housed in individual cages at 22–24 °C on a 12-h light–dark cycle. After 1-week adaptation period, rats were randomly divided into five groups (*n* = 10–12 per group): normal (N) group (*n* = 11) fed with a standard powder diet, aspirin (ASP) group (*n* = 10) fed with aspirin (700 mg/kg bw), LBP group (*n* = 12) fed with aspirin (700 mg/kg bw) and treated with LBP (100 mg/kg bw/d dissolved in 1 mL water) [21], CPC group (*n* = 12) fed with aspirin (700 mg/kg bw) and treated with CPC (50 mg/kg bw/d dissolved in 1 mL water) [22, 23], and MIX group (*n* = 11) fed with aspirin (700 mg/kg bw) and treated with LBP (50 mg/kg bw/d) + CPC (25 mg/kg bw/d). The dosage of LBP at 100 mg/kg bw/d showed antioxidant effects [21]. The dosage of CPC at 50 mg/kg bw/d had antioxidant and anti-inflammatory effects [22, 23]. The normal group was fed with a standard powder diet (Laboratory Rodent 5001 powder diet, PMI Nutrition International Inc., Brentwood, MO, USA) containing 48.7% (w/w) carbohydrate (58.0% total calorie), 23.9% (w/w) protein (28.5% total calorie), and 5.0% (w/w) fat (13.5% total calorie) for 9 weeks. The aspirin group was fed with aspirin (A5376, Sigma-Aldrich, St. Louis, MO, USA) which was mixed into the feed after 12-h fasting from week 2 to week 9 to develop gastric ulcer. The aspirin group and the treatment groups were given by daily gavage feeding of water or treatment, respectively, in a total volume of 1 mL at 9:00 am from week 1 to week 9, and aspirin was given by mixing into the feed 30-min after gavage feeding from week 2 to week 9. *Lycium barbarum* polysaccharides extract containing 40% polysaccharides were purchased from the commercial source (GojiMax®40%, GOJ-01-040POLYS, Priority Healthfood Cor., New Taipei, Taiwan), and obtained by water extraction. The total polysaccharides were determined spectrophotometrically.

The powder of LBP extract was suspended in deionized water, and the suspension was mixed with 8 M trifluoroacetic acid (1:1) at 110 °C for 4 h to hydrolyze polysaccharides into monosaccharides. The compositions and concentrations (μg/mg) of monosaccharides were determined using high pH anion exchange chromatography-pulsed amperometric detection (HPAEC-PAD) [24], and different monosaccharides were used as standards. The amount (μg/mg) of total monosaccharides before and after hydrolysis was measured by Academia Sinica (Taipei, Taiwan) using HPAEC-PAD, and total carbohydrate was determined by the difference in monosaccharides between after and before hydrolysis. The compositions of monosaccharides were expressed as the percentage of total carbohydrate, and mentioned previously as 92.04% glucose, 4.84% mannose, 1.82% galactose, 0.89% arabinose, and 0.41% rhamnose [24].

C-phycocyanin was generously supplied by Far East Bio-Tec. Co., Ltd. (Taipei, Taiwan), and contained 25% C-phycocyanin. The powder of *Spirulina platensis* was mixed with sterile water for 24 h, centrifuged to collect the supernatant, and dried by a freeze dryer. Water was sterilized at 121 °C for 30 min. The powder of C-phycocyanin contained 40% protein (62% C-phycocyanin and 38% other unidentified proteins), 25% polysaccharides, 6% nucleotides, 5% ash, 5% water, and 19% other unidentified ingredients. Feed intake was recorded daily, and body weight was measured weekly. After 9-week experimental period, rats were anesthetized by intraperitoneal injection with Zoletil 50 (1 mL/kg bw) and Rompun (0.5 mL/kg bw) after 12-h fasting and then sacrificed. Blood samples (*n* = 10–12 per group) were collected from the tail vein, allowed to be clotted at room temperature, and centrifuged at 1000 *g* at 4 °C for 10 min. Serum was separated in the supernatant and used for hydroxyproline analysis. The whole stomach tissue (*n* = 10–12 per group) was excised for ulcer assessment and biochemical analyses. The excised stomach tissue (5 mm × 5 mm) from the normal or lesion area was preserved in 10% buffered formalin for histopathological analysis. The remaining stomach tissue was aliquoted and stored at − 20 °C for further biochemical analysis. Gastric mucosa (*n* = 5 per group) was collected by scraping using a glass slide and used for total genomic DNA extraction in gastric microbiota analysis. All animal protocols were approved by the Institutional Animal Care and Use Committee at Taipei Medical University (LAC-2015-0214).

### Ulcer assessment

The formation of gastric ulcer was assessed pathologically using both ulcer index (UI) and ulcer score. The whole stomach tissue was harvested, opened along the greater curvature, and the mucosa was exposed for ulcer evaluation. The opened stomach tissue was fixed on the cork board with fine needles, and red coloration and hemorrhagic streaks in the ulcerated area were observed. The ulcerated area was assessed using planimetry under 1 mm × 1 mm graph paper with recording camera. Ulcer index was calculated using ImageJ software as below [25]:

$${\text{Ulcer}}\,{\text{index}}\, = \,{1}0/x,\quad {\text{where}}\quad x\, = \,{\text{total}}\,{\text{mucosal}}\,{\text{area}}/{\text{total}}\,{\text{ulcerated}}\,{\text{area}}.$$

The excised stomach tissue (5 mm × 5 mm) from the normal or lesion area was preserved in 10% buffered formalin overnight, embedded in paraffin wax, and stained with H&E to observe the damaged stomach. Coded specimens were evaluated by a pathologist in a blinded fashion. Ulcer score was evaluated under a light microscope according to the following criteria: 0: normal coloration, 0.5: red coloration, 1: spot ulcers, 1.5: hemorrhagic streaks, 2: ulcers > 3 mm but < 5 mm, and 3: perforation [25].

The development of gastric ulcer was monitored biochemically using serum hydroxyproline levels. Hydroxyproline has been considered as a serum biomarker for gastric ulcer in rats [26]. A decrease in serum hydroxyproline levels reflected the development of gastric ulcer by aspirin, alcohol, or stress in rats [26]. Serum hydroxyproline were assessed by an enzymatic colorimetric method using a commercial reagent kit (CSB-E08838r, Cusabio Biotech Co., Ltd., College Park, MD, USA), and the absorbance was measured at 450 nm and corrected at 540 nm.

### Gastroprotective factors in stomach tissue

Cyclooxygenase-1 (COX-1), cyclooxygenase-2 (COX-2), prostaglandin E_2_ (PGE_2_), and total nitrite and nitrate (NOx) levels as gastroprotective factors in stomach tissue were determined. The stomach tissue (100 mg) was homogenized in phosphate buffered saline (PBS) (1 mL), centrifuged at 5000*g* at 4 °C for 5 min, and the supernatant was collected for COX-1, COX-2, and PGE_2_ analyses. All analyses were measured by enzymatic colorimetric methods using commercial reagent kits (CSB-E1346r for COX-1, E3399r for COX-2, and E07967r for PGE_2_, Cusabio Biotech Co., Ltd., College Park, MD, USA), and the absorbance was analyzed at 450 nm and corrected at 540 nm for COX-1 [27] and COX-2 [28] and at 600 nm for PGE_2_ [29]. The stomach homogenate was centrifuge at 10,000*g* at 4 °C for 20 min, the supernatant was subsequently centrifuged at 100,000*g* for 30 min at 4 °C, and the supernatant was finally used for total nitrite and nitrate analysis. Gastric total nitrite and nitrate levels which represent the final metabolites of nitric oxide oxidation were assessed by an enzymatic colorimetric method using a commercial kit with Griess reagent (No 780001, Cayman Chemical, Ann Arbor, MI, USA), and the absorbance was measured at 540 nm [29]. All measurements are expressed as weight or concentration per mg protein in stomach tissue. Protein content in stomach tissue was determined by Bradford assay [30] using a commercial reagent kit (No 500-0113, Bio-Rad Laboratories, Inc., Hercules, CA, USA).

### Gastric antioxidant markers

The activity of superoxide dismutase (SOD) and the levels of lipid peroxides malondialdehyde (MDA) in stomach tissue were analyzed to determine antioxidant activity in different groups. The stomach tissue (100 mg) were homogenized with the buffer (1 mL) containing 20 mM 4-(2-hydroxyethyl)-1-piperazineethanesulfonic acid (HEPES), 1 mM ethylene glycol tetraacetic acid (EGTA), 210 mM mannitol, and 70 mM sucrose, centrifuged at 1500*g* for at 4 °C for 5 min, and the supernatant was used for SOD analysis. The activity of SOD was measured by an enzymatic colorimetric method using a commercial reagent kit (No 706002, Cayman Chemical, Ann Arbor, MI, USA) [31]. The stomach homogenate (10 μL) was mixed with xanthine oxidase (20 μL) for 30 min, and the absorbance was recorded at 450 nm.

The stomach tissue (100 mg) was homogenized with radioimmunoprecipitation assay buffer (1 mL), centrifuged at 1600*g* at 4 °C for 10 min, and the supernatant was analyzed for MDA levels. The levels of MDA were determined by an enzymatic colorimetric method [32] using a commercial reagent kit (No. 10009055, Cayman Chemical, Ann Arbor, MI, USA). The stomach homogenate (10 μL) was added with sodium dodecyl sulfate (10 μL) and color reagent (400 μL) containing 2-thiobarbituric acid and acetic acid, and incubated at 90–100 °C for 60 min. The samples were put in ice for 10 min to stop the reaction, centrifuged at 1600*g* at 4 °C for 10 min, put at room temperature for 30 min, and the absorbance was measured at 540 nm.

### Protein expression of inflammatory markers in stomach tissue

The inflammatory markers such as nuclear factor (NF)-κB (P65), intercellular adhesion molecule (ICAM)-1, tumor necrosis factor (TNF)-α, interleukin (IL)-1β, and IL-10 in stomach tissue were measured by enzyme-linked immunosorbent assays. The stomach tissue (100 mg) was homogenized in PBS (1 mL), mixed with complete hypotonic buffer (50 μL) and nonyl phenoxypolyethoxylethanol (NP-40) (5 μL), and centrifuged at 14,000*g* at 4 °C for 30 s. The supernatant was mixed with complete nuclear extraction buffer (10 μL) on ice for 30 min, centrifuged at 14,000*g* at 4 °C for 10 min, and the supernatant as analyzed for NF-κB (P65) binding to DNA using a commercial reagent kit (No 10007889, Cayman Chemical, Ann Arbor, MI, USA) [33]. The samples were added complete transcription factor binding assay buffer at 4 °C overnight, washed in wash buffer for several times, incubated with NF-κB (P65) primary antibody for 1 h, and then added goat anti-rabbit IgG horseradish peroxidase (HRP) conjugate secondary antibody for 1 h with several washes in between. Finally, the samples were added transcription factor developing solution in dark for 30 min and mixed with stop solution (H_2_SO_4_), and the absorbance was recorded at 450 nm.

The levels of ICAM-1 in stomach tissue were determined using a commercial reagent kit (CSB-E04576r, Cusabio Biotech Co., Ltd., College Park, MD, USA) [34]. The stomach homogenate (100 μL) in PBS was added to a 96-well plate with pre-coated primary antibody at 37 °C for 2 h, incubated with biotin-conjugated antibody (100 μL) specific to ICAM-1 at 37 °C for 1 h, and then added HRP-avidin (100 μL) at 37 °C for 1 h after several washes. The samples were added substrate solution (3,3′,5,5′-tetramethylbenzidine, TMB) in dark at 37 °C for 15 min, and then added stop solution (acetic acid). The absorbance was measured at 450 nm and corrected at 540 nm.

The levels of TNF-α, IL-1β, and IL-10 in stomach tissue were assessed using commercial reagent kits (DY510 for TNF-α, DY501 for IL-1β, DY522 for IL-10, R&D Systems, Inc., Minneapolis, MN, USA) [35]. The stomach homogenate (100 μL) in PBS was added to a 96-well plate with pre-coated primary antibody for 2 h, mixed with detection antibody (100 μL) for 2 h, and then incubated with streptavidin-HRP (100 μL) in dark for 20 min with several washes in between. The samples were added TMB substrate solution (100 μL) for 20 min, and then added stop solution (50 μL 2 N H_2_SO_4_). The absorbance was determined at 450 nm and corrected at 540 nm.

### Taxonomic compositions and diversity of gastric microbiota

The gastric microbiota composition before and after treatments (*n* = 5 per group) was profiled using next-generation sequencing (NGS) approach. Total genomic DNA in gastric mucosa was extracted and purified by a commercial kit (No 51704, QIAGEN GmbH, Hilden, Germany). The 16S rRNA gene amplicons were generated by domain-level polymerase chain reaction (PCR) from genomic DNA for the Illumina MiSeq System. The V3-V4 region of the bacterial 16S rRNA genes was amplified by PCR using 16S rRNA primers (forward primer 341F: 5′-CCTACGGGNGGCWGCAG-3′, reverse primer 805R: 5′-GACTACHVGGGTATCTAATCC-3′). The PCR was performed by denaturation at 95 °C for 30 s, annealing at 55 °C for 30 s, and elongation at 72 °C for 30 s for 25 cycles. The amplified product was ligated into a sequencing primer, and a sequencing library was constructed followed by NGS sub-generation sequencing genotype analysis using an Illumina MiSeq sequencer. The microbial strains were identified using the SILVA database (v128) [36], and multiple sequence alignment analysis of microbial strains was performed by Decipher package (v2.2.0). The phylogenetic tree was constructed from the alignment using phangorn package (v2.2.0) [37]. The taxonomy assignment and phylogenetic tree were consolidated into phyloseq package, and community analyses were conducted using phyloseq package (v1.19.1) [38]. The sequence fragment reads were classified, and the resulting counts of classified reads were used to estimate relative abundance.

Diversity of gastric microbiota was determined by alpha and beta diversity. The alpha diversity represents the differences within a microbial ecosystem to estimate how many different species could be detected in a microbial ecosystem (species richness) and how different the microbes are distributed in a microbial ecosystem (species diversity). Species richness was estimated by observed and Chao1 indices, and species diversity was determined by Shannon and Simpson’s indices using phyloseq package. The beta diversity indicates the differences between microbial communities from different environments to estimate how different the microbial composition is in one environment compared with another. The UniFrac distance is a distance metric for comparing biological communities, and was calculated using GUniFrac package (v1.1) to evaluate the community dissimilarity between different groups [39]. Principal coordinate analysis (PCoA) ordination on unweighted and weighted UniFrac distance was conducted using the adonis and betadisper functions of the vegan package (v2.4, https://CRAN.R-project.org/package=vegan) to analyze the dissimilarity of composition among different groups and the homogeneity of dispersion, respectively. Comparisons of microbiota enrichment were analyzed to screen bacterial species which are most likely to explain the differences among different groups using the linear discriminant analysis (LDA) effect size (LEfSe) method. The results were visualized as a cladogram using GraPhlAn [40]. The logarithmic LDA score > 2 is considered significantly different at *p* < 0.05 [41].

### Statistical analysis

Data are expressed as mean ± standard error of means (SEM). The mean differences in body weight, food intake, ulcer assessment, and biochemical parameters among different groups (*n* = 10–12 per group) were analyzed by one-way analysis of variance (ANOVA) followed by Fisher’s least significant difference test. Comparisons of the differences in relative abundance, alpha diversity, and beta diversity of gastric microbiota among different groups (*n* = 5 per group) were performed by Kruskal–Wallis one-way ANOVA followed by Wilcoxon-Mann–Whitney test. All statistical analyses were performed using SAS 9.4 (SAS Institute Inc., Cary, NC, USA). Statistical significance is assigned at *p* < 0.05.

## Results

### Body weight, food intake, and aspirin intake

There were no significant differences in body weight at weeks 0 and 1 among different groups (Table 1). Body weight of rats in all aspirin-treated groups was significantly decreased by 14%–19% compared with that in the normal group at week 9 (*p* < 0.05). Body weight gains from week 0 to week 9 were reduced in all aspirin treated groups compared with those in the N group (*p* < 0.05). Accumulated body weight gains are shown in Additional file 1: Fig. S1. Body weight gains were decreased at week 2 in all the groups compared with those at week 1 due to aspirin feeding at week 1. Body weight gains were gradually increased after week 2 in the N group, and significantly higher from week 3 to week 9 compared with those in the ASP group (*p* < 0.05). However, body weight gains had delayed to be elevated until week 3 in all aspirin treated groups. The average daily food intake of rats was 29–31 g and 19–22 g during the pre-treatment period at week 1 and the ulcer development period from week 2 to week 9, respectively. There was no significant difference in food intake among different groups at week 1. However, food intake in all aspirin treated groups was significantly reduced compared with that in the normal group (*p* < 0.05). Additionally, food intake in the LBP treated group was significantly increased by 7% compared with that in the ASP group (*p* < 0.05). Aspirin intake completion rate is the percentage of aspirin actual intake to the assigned dosage of aspirin. The completion rate of aspirin intake was 84%-90% in different groups. Corresponding to an increase in food intake by 7% in the LBP group, both aspirin intake and completion rate in the LBP group were significantly increased by 7% compared with those in the ASP group (*p* < 0.05).

**Table 1:** Body weight, food intake, and aspirin intake of rats in different groups

	N	ASP	LBP	CPC	MIX
Body weight (g/d)					
Week 0	229 ± 2	232 ± 2	231 ± 2	234 ± 2	233 ± 2
Week 1	305 ± 2	305 ± 2	307 ± 1	308 ± 3	310 ± 3
Week 9	427 ± 7	344 ± 11*	368 ± 7*	352 ± 11*	351 ± 8*
Weight gain (g)	197.3 ± 6.9	111.9 ± 11.1*	137.6 ± 7.1*	117.4 ± 11.1*	118.5 ± 8.4*
Food intake (g/d)					
Pre-treatment (week 1)	29.4 ± 0.3	29.4 ± 0.5	30.5 ± 0.2	30.2 ± 0.2	29.9 ± 0.2
Induction (weeks 2–9)	21.9 ± 0.3	18.5 ± 0.5*	19.8 ± 0.2*^#^	18.9 ± 0.4*	18.5 ± 0.4*
Aspirin intake (mg/kg bw/d)	–	587 ± 16	628 ± 8^#^	601 ± 13	590 ± 14
Completion rate (%)	–	84 ± 2	90 ± 1^#^	86 ± 2	84 ± 2

Data are mean ± SEM (*n* = 10–12 per group). Differences between the groups were determined by one-way ANOVA followed by Fisher’s least significant difference test

N, standard powder diet; ASP, aspirin; LBP, aspirin + *Lycium barbarum* polysaccharides (LBP); CPC, aspirin + C-phycocyanin (CPC); MIX, aspirin + LBP + CPC

*p < 0.05 compared with the N group; ^#^*p* < 0.05 compared with the ASP group

### Effects of LBP and/or CPC on ulcer index and serum hydroxyproline

**Fig. 1:**
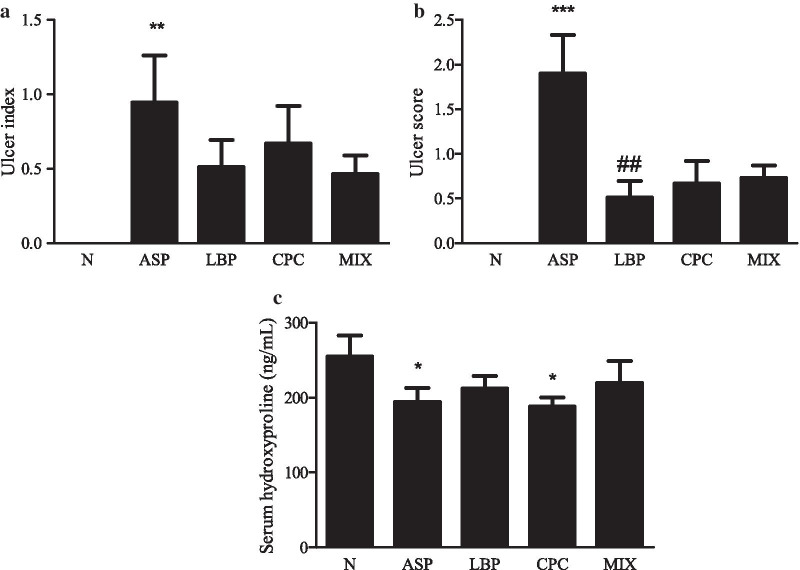
Effects of *Lycium barbarum* polysaccharides (LBP) and/or C-phycocyanin (CPC) on **a** ulcer index, **b** ulcer score, and **c** serum hydroxyproline level in stomach tissue. N, standard powder diet; ASP, aspirin; LBP, aspirin + *Lycium barbarum* polysaccharides (LBP); CPC, aspirin + C-phycocyanin (CPC); MIX, aspirin + LBP + CPC. Data are mean ± SEM (*n* = 10–12 per group). Differences between the groups were determined by one-way ANOVA followed by Fisher’s least significant difference test. *p < 0.05 compared with the N group; **p < 0.01 compared with the N group; ***p < 0.001 compared with the N group; ^##^*p* < 0.01 compared with the ASP group

Red coloration and hemorrhagic streaks in the gastric mucosal surface were observed for the formation of ulcer in different groups. There was no redness and swelling on the gastric surface in the N group, however, the hemorrhagic streaks were obvious in the ASP group, and redness was observed in the LBP, CPC, and MIX groups (Additional file 1: Fig. S2a). The histological staining of stomach tissue is shown in Additional file 1: Fig. S2b, and corresponding ulcer scores are indicated in Fig. 1b. The ASP group had significantly higher ulcer index (*p* < 0.001) and ulcer score (*p* < 0.01) than the N group (Fig. 1a). The LBP group had significantly lower ulcer score than the ASP group (0.5 ± 0.2 vs. 1.9 ± 0.2, *p* < 0.01) (Fig. 1b). There were no significant differences in ulcer index between the LBP and/or CPC treated groups and the ASP group (Fig. 1a). However, the LBP (*p* = 0.05) and MIX groups (*p* = 0.07) tended to reduce ulcer index compared with the ASP group (Fig. 1a). Serum hydroxyproline levels as a biochemical index of gastric ulcer were significantly decreased by 24% and 26% in the ASP and CPC groups, respectively, compared with the N group (*p* < 0.05) (Fig. 1c).

### Effects of LBP and/or CPC on gastroprotective factors in stomach tissue

**Fig. 2:**
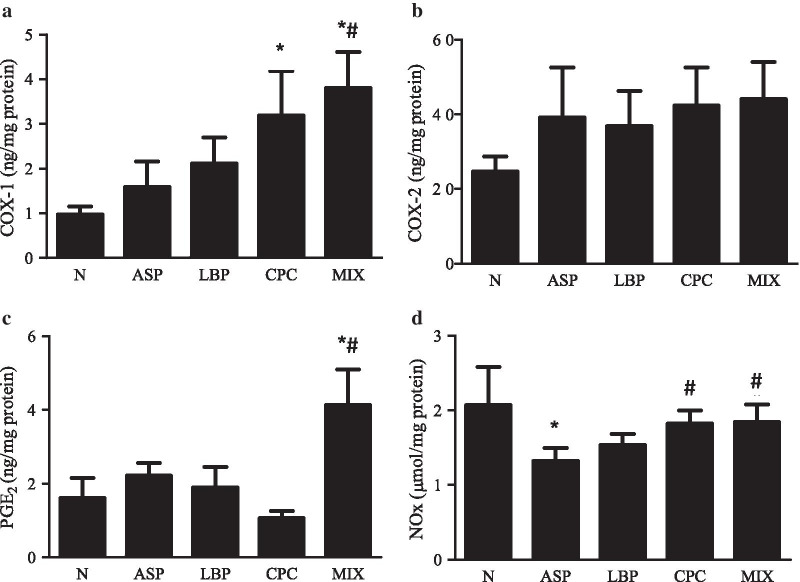
Effects of *Lycium barbarum* polysaccharides (LBP) and/or C-phycocyanin (CPC) on the levels of **a** cyclooxygenase-1 (COX-1), **b** cyclooxygenase-2 (COX-2), **c** prostaglandin E_2_ (PGE_2_), and **d** total nitrite and nitrate (NOx) in stomach tissue. N, standard powder diet; ASP, aspirin; LBP, aspirin + *Lycium barbarum* polysaccharides (LBP); CPC, aspirin + C-phycocyanin (CPC); MIX, aspirin + LBP + CPC. Data are mean ± SEM (*n* = 10–12 per group). Differences between the groups were determined by one-way ANOVA followed by Fisher’s least significant difference test. *p < 0.05 compared with the N group; ^#^*p* < 0.05 compared with the ASP group

Gastric COX-1 levels were significantly elevated by 2.3 and 2.9 times in the CPC and MIX groups, respectively, compared with those in the N group (*p* < 0.05) (Fig. 2a). The MIX group had higher gastric COX-1 levels than the ASP group (3.80 vs. 1.59 ng/mg protein) (*p* < 0.05). Gastric COX-2 levels did not differ among different groups (Fig. 2b). Gastric PGE_2_ levels were significantly increased by 155% or 86% in the MIX group compared with the N or ASP group, respectively (*p* < 0.05) (Fig. 2c). Gastric total nitrite and nitrate levels were significantly reduced by 38% in the ASP group compared with the N group (*p* < 0.05) (Fig. 2d). Both CPC and MIX groups significantly increased gastric total nitrite and nitrate levels by 64% and 66%, respectively, compared with the ASP group (*p* < 0.05).

### Effects of LBP and/or CPC on gastric antioxidant markers

**Fig. 3:**
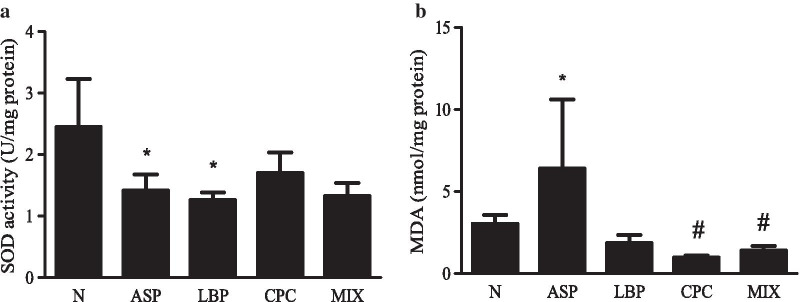
Effects of *Lycium barbarum* polysaccharides (LBP) and/or C-phycocyanin (CPC) on gastric antioxidative markers. **a** Superoxide dismutase (SOD) activity and **b** malondialdehyde (MDA) levels. N, standard powder diet; ASP, aspirin; LBP, aspirin + *Lycium barbarum* polysaccharides (LBP); CPC, aspirin + C-phycocyanin (CPC); MIX, aspirin + LBP + CPC. Data are mean ± SEM (*n* = 10–12 per group). Differences between the groups were determined by one-way ANOVA followed by Fisher’s least significant difference test. *p < 0.05 compared with the N group; ^#^*p* < 0.05 compared with the ASP group

Gastric SOD activity in the ASP and LBP groups were significantly decreased by 42% and 49%, respectively, compared with that in the N group (*p* < 0.05) (Fig. 3a). However, there were no significant differences in gastric SOD activity among the ASP treated groups. Gastric MDA levels in the ASP groups were significantly increased by 1.1 times compared with those in the N group (*p* < 0.05) (Fig. 3b). The CPC and MIX groups significantly reduced gastric MDA levels by 85% and 78%, respectively, compared with the ASP group (*p* < 0.05), and the LBP group tended to decrease gastric MDA levels compared with the ASP group (*p* = 0.06).

### Effects of LBP and/or CPC on gastric inflammatory markers

There were no significant differences in gastric NF-κB (p65) activity, ICAM-1, TNF-α, IL-1β, and IL-10 levels (Additional file 1: Fig. S3) among different groups.

### Effects of LBP and/or CPC on taxonomic compositions and diversity of gastric microbiota

**Fig. 4:**
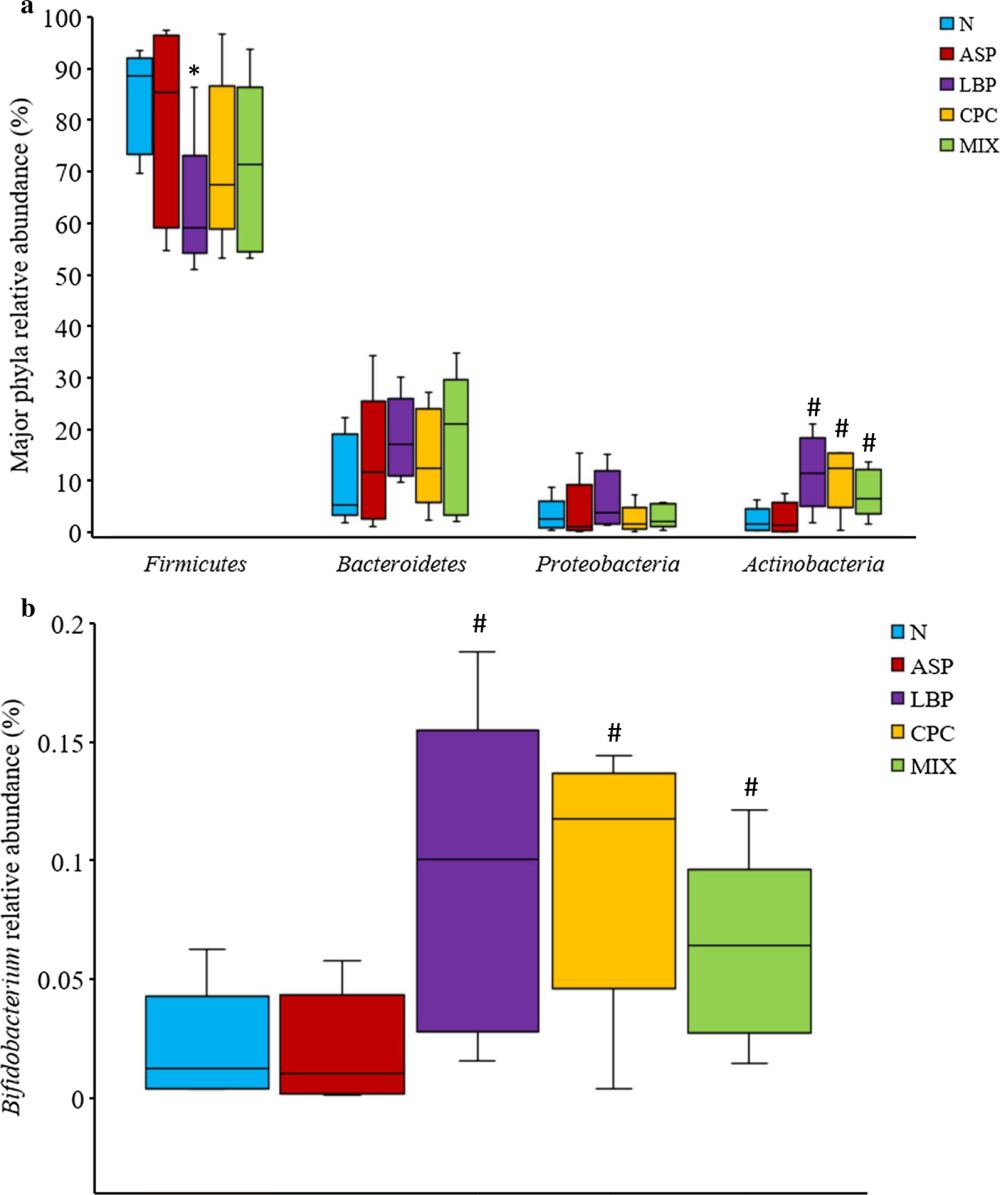
Effects of *Lycium barbarum* polysaccharides (LBP) and/or C-phycocyanin (CPC) on relative abundance of **a** major phyla and **b** *Bifidobacterium* genus in gastric microbiota. N, standard powder diet; ASP, aspirin; LBP, aspirin + *Lycium barbarum* polysaccharides (LBP); CPC, aspirin + C-phycocyanin (CPC); MIX, aspirin + LBP + CPC. The horizontal lines in a boxplot from the bottom to the top represent the 25th percentile, median, and the 75th percentile of diversity values in each group (*n* = 5 per group). Relative abundance (%) of major phyla is percentage distribution of bacteria in the classification to all microbiota. Differences between the groups were determined by Kruskal–Wallis one-way ANOVA followed by Wilcoxon-Mann–Whitney test. *p < 0.05 compared with the N group; ^#^*p* < 0.05 compared with the ASP group

The taxa distribution of rat gastric microbiota by phylum, class, family, and genus is shown in Additional file 1: Fig. S4. *Firmicutes*, *Bacteroidetes*, *Proteobacteria*, and *Actinobacteria* were the major phyla in gastric microbiota in the N or ASP treated groups (Fig. 4a, Additional file 1: Fig. S4a). The relative abundance of the phylum *Firmicutes* in the LBP group was significantly decreased compared with the N group (*p* < 0.05) (Fig. 4a). However, the relative abundance of the phylum *Actinobacteria* in the LBP, CPC, and MIX groups were significantly increased compared with the ASP group (*p* < 0.05). The relative abundance of the phyla *Bacteroidetes* and *Proteobacteria* was not significantly different among different groups. The relative abundance of the genus *Bifidobacterium* in the LBP, CPC, and MIX groups was significantly elevated by 3.5, 4.0, and 2.5 times, respectively, compared with the ASP group (*p* < 0.05) (Fig. 4b). There were no significant differences in the relative abundance of the genus *Bifidobacterium* between the N and ASP groups. The relative abundance of the genus *Streptococcus* was not significantly different between any two groups (data were not shown).

**Fig. 5:**
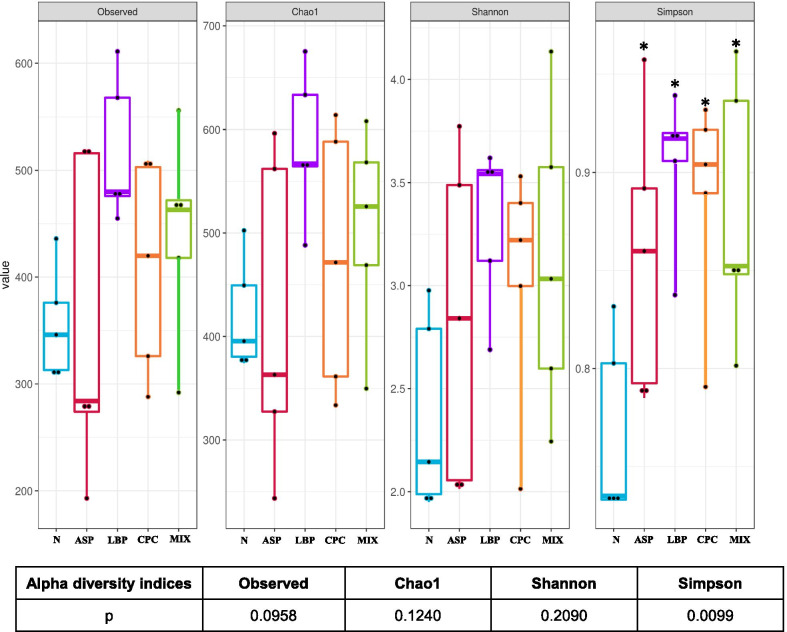
Alpha diversity of gastric microbiota. Species richness was measured by observed and Chao1 indices. Species diversity was determined by Shannon and Simpson’s indices. N, standard powder diet; ASP, aspirin; LBP, aspirin + *Lycium barbarum* polysaccharides (LBP); CPC, aspirin + C-phycocyanin (CPC); MIX, aspirin + LBP + CPC. The horizontal lines in a boxplot from the bottom to the top represent the 25th percentile, median, and the 75th percentile of diversity values in each group (*n* = 5 per group). The bottom and top solid dots indicate the minimum and maximum values, respectively. The *p* value < 0.05 is considered statistically significant using Kruskal–Wallis one-way ANOVA. *p < 0.05 compared with the N group by Wilcoxon-Mann–Whitney test

The alpha diversity was determined by species richness (observed and Chao1 indices) and species diversity (Shannon and Simpson’s indices). Only Simpson’s indices were significantly different among different groups (Fig. 5). The Simpson’s index values were significantly higher in all the ASP treated groups compared with the N group (*p* < 0.05). The beta diversity of gastric microbiota is indicated in Additional file 1: Fig. S5. The beta diversity of gastric microbiota in unweighted and weighted UniFrac distances was not significantly different among five groups using Kruskal–Wallis one-way ANOVA (*p* > 0.05).

**Fig. 6:**
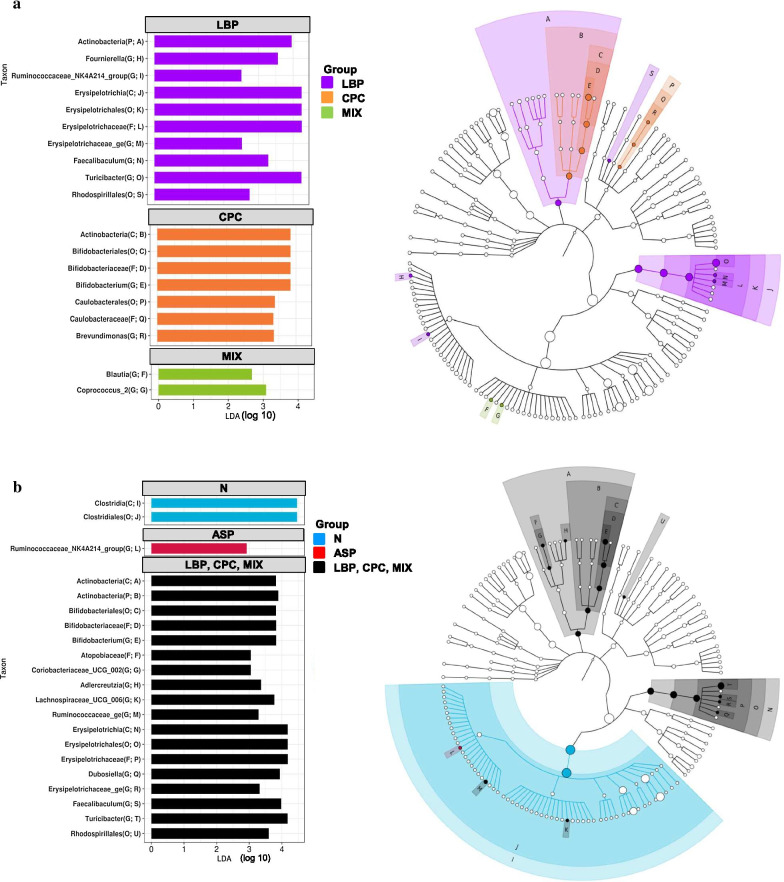
Gastric microbiota enrichment in different groups (*n* = 5 per group) using the linear discriminant analysis (LDA) effect size (LEfSe). **a** Gastric microbiota enrichment in each group and **b** gastric microbiota enrichment in the N, ASP, and treated groups (LBP, CPC, and MIX). N, standard powder diet; ASP, aspirin; LBP, aspirin + *Lycium barbarum* polysaccharides (LBP); CPC, aspirin + C-phycocyanin (CPC); MIX, aspirin + LBP + CPC. C, class; F, family; G, genus; O, order; P, phylum. The circles of phylogenetic tree cladogram in the right panel from inside to outside represent the classification level from phylum to genus of gastric microbiota. The diameter of the circle indicates the relative abundance. The letters A to S in **a** and A to U in **b** represent 19 and 21 different taxa, respectively. The abbreviations in the parentheses in the left panel are taxonomy rank and position on the cladogram in the right panel, respectively. The logarithmic LDA score > 2 shown in the left panel is considered significantly different at *p* < 0.05. There were no significant differences in LDA scores in the N or ASP group compared with three treated groups in **a**

Gastric microbiota enrichment in different groups is shown in Fig. 6 using the linear discriminant analysis (LDA) effect size (LEfSe) method. A total of 19 significant enrichment taxa with LDA score > 2 in the treated groups were found (Fig. 6a). There were no significant differences in LDA scores in the N or ASP group compared with 3 treated groups. The LBP group had 10 significant enrichment taxa including the phylum *Actinobacteria*, the class *Erysipelotrichia*, the order *Erysipelotrichales*, the family *Erysipelotrichaceae*, and the genus *Turicibacter* of the phylum *Firmicutes*. The CPC group had 7 significant enrichment taxa including the class *Actinobacteria*, the order *Bifidobacteriales*, and the family *Bifidobacteriaceae* of the phylum *Actinobacteria*. The MIX group had 2 significant enrichment taxa including the genera *Blautia* and *Coprococcus* of the phylum *Firmicutes*. As compared with the ASP or treated groups (LBP, CPC, and MIX groups together), the N group had significant enrichment in the class *Clostridia* and the order *Clostridiales* of the phylum *Firmicutes* (Fig. 6b). The ASP group had significantly higher in genus *Ruminococcaceae* of the phylum *Firmicutes* compared with the N or treated groups. The treated groups had significantly higher in the phylum and class *Actinobacteria*, the order *Bifidobacteriales*, the family *Bifidobateriaceae*, and the genera *Bifidobacterium*, *Coriobacteriaceae*, and *Adlercreutzia* of the phylum *Actinobacteria* compared with the N or ASP group.

## Discussion

Our study showed that oral administration of aspirin (700 mg/kg bw) for 8 weeks led to the development of gastric ulcer. Similarly, a previous study found that serum hydroxyproline levels were significantly reduced in rats with aspirin-induced gastric ulcer by a single oral administration (300 mg/kg bw) [42]. Aspirin could decrease the gastroprotective factor such as total nitrite and nitrate levels in the stomach and increase oxidative stress by decreasing gastric SOD activity and increasing gastric lipid peroxides to result in the development of gastric ulcer. Although the treated groups had the tendency to decrease gastric ulcer index and ulcer scores compared with the ASP group, only the LBP group significantly decreased gastric ulcer score. However, the LBP group did not increase serum hydroxyproline levels after 9-week treatment period. The reason for not significant difference in gastric ulcer index and ulcer scores by LBP and/or CPC treatments was speculated that the 9-week treatment period might be too short to observe complete healing of gastric ulcer caused by aspirin in terms of pathological observation. The clinical therapy for the patients with *Helicobacter pylori*-infected peptic ulcer took 10–14 days to eradicate *Helicobacter pylori* [43]. For the patients with dyspepsia but no alarm symptoms such as bleeding, perforation, obstruction, and malignancy in the gastrointestinal tract, antisecretory therapy such as proton pump inhibitors and histamine H_2_ blockers was administered for 4–8 weeks [44]. However, up to 25% of gastric ulcer patients who continuously took NSAIDs during therapy needed more than 8 weeks for proton pump inhibitor therapy [43].

Serum hydroxyproline levels as a biomarker for gastric ulcer were significantly decreased by CPC treatment in the present study. Similarly, a previous study found that CPC reduced lung hydroxyproline levels as a biomarker for collagen deposition or fibrosis and had anti-fibrotic effects on bleomycin-induced pulmonary fibrosis in mice by mediating toll-like receptor 2 signaling pathway [44]. It is hypothesized that gastric mucosal healing could be affected by CPC due to its anti-fibrotic effects, and gastric ulcer score did not significantly decreased by CPC.

Our data showed that chronic oral administration of aspirin did not significantly change gastric COX-1, COX-2, and PGE_2_ levels, but significantly reduced gastric total nitrite and nitrate levels. Aspirin is a more potent inhibitor of COX-1 than of COX-2, and the synthesis of prostaglandins could be altered due to the changes in COX-1 and/or COX-2 activities [45]. Prostaglandins synthesized by COX-1 can protect the gastric mucosa against damage, and these synthesized by COX-2 can stimulate inflammatory reaction, suggesting that both COX-1 and COX-2 might contribute to gastric mucosal defense [45]. Gastric PGE_2_ levels were not significantly altered by aspirin in this study probably because of the change in neither COX-1 nor COX-2 levels. Consistent with our findings, a previous study revealed that gastric ulcer caused by a single oral dose (400 mg/kg bw) of aspirin significantly decreased gastric total nitrite and nitrate levels, indicating gastric protection from the production of NO was reduced [10].

The MIX group significantly elevated gastric COX-1 and PGE_2_ levels, and the CPC and MIX groups also significantly increased gastric total nitrite and nitrate levels, suggesting that the combination of CPC and LBP can increase the gastroprotective factors to further protect against gastric ulcer. The previous study revealed that inhibition of NO production by NO synthase inhibitors impaired the gastroprotective effect of modafinil on the healing of gastric lesion induced by indomethacin or water-immersion stress in rats, suggesting that modulation of NO pathway could be potentially crucial for gastroprotection [46]. The possible mechanisms for the gastroprotective effect of NO against gastric ulcer could be associated with regulation of mucosal blood flow, increases in COX activity and PGE_2_ synthesis, inhibition of neutrophil infiltration, and maintenance of mucus secretion by goblet cells [46]. However, LBP did not increase gastric total nitrite and nitrate levels. It is speculated that gastric pH value could be affected by the production of short-chain fatty acids via the fermentation of LBP by gastric microbiota [19]. The altered gastric pH could weaken the potential role of LBP for the protection against gastric ulcer.

Aspirin inhibited gastric SOD activity and increased gastric lipid peroxides MDA levels. Oxidative stress is considered to be involved in the pathogenesis of gastric ulcer, and SOD as an oxidative enzyme scavenges reactive oxygen species [47]. Consistent with our results, gastric lipid peroxidation was increased in rats with aspirin- or alcohol-induced gastric mucosal injury [48–50]. The CPC and MIX groups significantly reduced gastric MDA levels, and the LBP group tended to decrease gastric MDA levels. A previous study found that LBP supplement (100 mg/kg bw) for 24 weeks activated hepatic nuclear factor erythroid 2-related factor 2 (Nrf2) and reduced intracellular reactive oxygen species in C57BL/6 J mice with high fat diet-induced insulin resistance [51]. Therefore, LBP as an antioxidant [52] can attenuate oxidative stress via activating Nrf2 to inhibit oxidative stress-induced damage [53]. Whereas CPC as an antioxidant could also scavenge free radicals and inhibit microsomal and CCl_4_-induced lipid peroxidation in vitro and in vivo, respectively [54].

Aspirin or treatments did not change protein expression of gastric pro-inflammatory or anti-inflammatory markers in our study. A previous study showed that oral administration of aspirin in a single dose of 400 mg/kg bw significantly increased gastric TNF-α, IL-6, and IL-12 levels, but significantly decreased gastric IL-4 and IL-10 levels in rats with gastric mucosal damage caused by aspirin after 8 h of aspirin administration [10]. Similarly, oral administration of aspirin in a single dose of 200 mg/kg bw significantly increased serum TNF-α and IL-1β levels in rats with gastric ulcer caused by aspirin after 4 h of aspirin administration [54]. The different results from our findings could be due to different duration of aspirin administration. In our study, oral administration of aspirin was chronic and continuous for 8 weeks, and the inflammatory response could not be increased after chronic use of aspirin because of the anti-inflammatory effect of aspirin. In the previous studies, gastric mucosa damage was caused by a single administration of aspirin and rats were sacrificed in a short time after aspirin administration [10, 55]. Further studies are needed to evaluate whether mRNA expression of inflammatory markers could be regulated by aspirin or treatments at the transcriptional level.

The *Firmicutes* phylum was the most abundant phylum in gastric microbiota of rats with gastric ulcer caused by aspirin. The aspirin-treated groups tended to increase the relative abundance of the genus *Streptococcus* compared with the N group (3.7 ± 3.5 vs. 0.4 ± 0.1%, *p* > 0.05), but there were no significant differences among five groups due to a large variation. Similarly, the *Firmicutes* phylum and the *Streptococcus* genus in the gastric biopsies from the patients with antral gastritis were more relative abundance compared with those in the normal biopsies [56]. *Clostridium difficile* in the *Firmicutes* phylum was related to the inflammatory response and bowel disease [57]. The patients with *Clostridium difficile*-associated diarrhea had significantly elevated mean platelet volume, neutrophil–lymphocyte ratio, and serum C-reactive protein levels compared with age- and sex-matched subjects with diarrhea but without *Clostridium difficile* infection [57]. However, whether the increase in the relative abundance of *Firmicutes* is causative for gastric ulcer or as a result of microbial changes due to gastric ulcer was remained to be further investigated. Consistent with our data, *Firmicutes*, *Bacteroidetes*, *Proteobacteria*, and *Actinobacteria* were predominant phyla in normal rat gastric microbiota [58]. At the genus level, *Turicibacter*, *Lactobacillus*, and *Clostridium* of the phylum *Firmicutes* were relatively abundant in normal rat gastric microbiota [58]. After aspirin administration, the genus *Ruminococcaceae* of the phylum *Firmicutes* was significantly enriched in rat stomach compared with the N or treated groups in our study. A previous study showed that family *Ruminococcaceae*, *Prevotella* species, *Bacteroides* species, and *Barnesiella* species were significantly discriminated in colonic microbiota of the adults using aspirin compared with those using no medication [59]. However, our study found that the relative abundance of the *Firmicutes* phylum in rat stomach was not significantly different among different groups except for the LBP group with lower relative abundance of the *Firmicutes* phylum as compared with the N group.

Our results revealed that supplementation of LBP and/or CPC significantly increased the relative abundance of the genus *Bifidobacterium* in the *Actinobacteria* phylum. It is suspected that increased relative abundance of the genus *Bifidobacterium* in the LBP, CPC, and MIX groups could be associated with the changes in gastroprotective factors. However, no significant associations were observed between the relative abundance of the genus *Bifidobacterium* and the levels of gastroprotective factors in the stomach (Additional file 1: Table S1). Increased *Bifidobacterium* by both LBP and CPC could be a source of potential beneficial bacteria in the stomach for the host’s health. The genus *Bifidobacterium* had protective effects on damaged gastric or intestinal mucosa in rats [60, 61] and humans [62]. Prophylactic administration of *Bifidobacterium animalis* VKL and VKB mixture for 14 days effectively protected against the development of ulcerative lesions in gastric mucosa caused by water immersion restraint stress in rats via preventing the degradation of mucus barrier [60]. The incidence of ileal ulcer developed by 5-bromo-2-(4-fluorophenyl)-3-(4-methylsulfonylphenyl) thiophene (BFMeT, a NSAID) in rats was decreased by oral administration of *Bifidobacterium adolescentis* in drinking water through inhibiting the growth of aerobic bacteria in the ileum and decreasing the production of lipid peroxides in ileal mucosa [61]. Daily oral administration of *Bifidobacterium breve* capsules (≥ 5 × 10^10^ colony-forming units) for 8 weeks significantly decreased the area under the curve for small intestinal damage (Lewis score) in adults with small intestinal lesions caused by 6-week oral intake of aspirin compared with those taking placebo without *Bifidobacterium breve* [62]. It is known that symbiotic bacteria in the intestine can break down non-digestible carbohydrates such as non-starch polysaccharides, resistant starch, oligosaccharides, and disaccharides, and further ferment these carbohydrates to release short-chain fatty acids [63]. These short-chain fatty acids produced by intestinal microbiota can be an energy source and decrease the luminal pH value, which is beneficial to the growth of probiotics and adverse to the growth of pathogens in the intestine [63]. An in vitro study found that LBP extract containing polysaccharides and polyphenols could be used as a source of food or nutraceuticals to help the growth of probiotics and protect the viability of *Bifidobacterium* and *Lactobacillus* in the simulated gastric and intestinal juices [64]. A previous study showed that LBP with major monosaccharides as glucuronic acid, galacturonic acid, glucose, galactose, and arabinose stimulated the production of short-chain fatty acids and increased the relative abundances of genera *Bifidobacterium*, *Bacteroides*, *Phascolarctobacterium*, *Clostridium* XlVb, *Prevotella*, and *Collinsella* in vitro after 24-h fermentation of LBP by healthy human fecal microbiota [19].

Supplementation of spirulina in vitro significantly promoted the growth of the lactic acid producing bacteria [65] because of the nitrogenous substance in spirulina [66]. Polyphenolic compounds in spirulina were also reported to regulate gut microbial communities [67]. In addition, oral ingestion of a dairy product containing *Lactobacillus* GG, *Lactobacillus helveticus*, and *Lactobacillus acidophilus* at a dose of 2.4 × 10^9^/strain/d for 5 days protected the integrity of the gastric mucosal barrier against the damage of indomethacin in healthy humans [68]. Our data indicated that CPC (a bioactive ingredient of spirulina) treatment significantly increased the class *Actinobacteria*, the order *Bifidobacteriales*, the family *Bifidobacteriaceae*, and the genus *Bifidobacterium* in the stomach. A recent study showed that daily oral pretreatment of phycocyanin (50 mg/kg bw) for 30 days increased the proportion of beneficial bacteria such as the genera *Lactobacillus*, *Bifidobacterium*, and *Roseburia* and decreased that of harmful bacteria such as the genus *Desulfovibrio* in the phylum *Proteobacteria* in the cecum and feces of mice with radiation-induced acute gastrointestinal syndrome [20]. The pretreatment of phycocyanin improved ileal integrity via increasing tight junction proteins and decreased intestinal inflammatory signaling proteins in mice with radiation-induced acute gastrointestinal syndrome [20]. In addition, the abundance of the genera *Roseburia*, *Enterorhabdus*, *Alistipes*, *Coriobacteriaceae*, *Lachnospiraceae*, and *Ruminococcaceae* in the cecum and feces was negatively associated with histopathological indicators of intestinal injury, but positively correlated with the levels of tight junction proteins [20]. It is suggested that CPC could modulate gastric microbiota to increase beneficial bacteria, which might be associated with enhancing intestinal integrity. Therefore, LBP and/or CPC may protect against gastric ulcer caused by NSAIDs via improving the compositions of gastric microbiota.

The relative abundance of the phylum *Bacteroidetes* in the LBP and MIX groups tended to be slightly increased, but was not significantly different among five groups. The phyla *Proteobacteria*, *Bacteroidetes*, and *Firmicutes* were predominant in patients with *Helicobacter pylori*-positive gastric antrum ulcer or duodenal ulcer [69]. The phylum *Proteobacteria* was mostly abundant in gastric antrum of the patients with *Helicobacter pylori*-positive gastric antrum ulcer, and the phyla *Bacteroidetes* and *Firmicutes* were mostly abundant in the duodenum of patients with *Helicobacter pylori*-positive duodenal ulcers [69]. Our data showed that the phyla *Firmicutes* and *Bacteroidetes* were predominant in aspirin treated groups. The proportion of bacteria in the phylum was different between humans and rats and between gastric ulcer and duodenal ulcer. In addition, the relative abundance of bacteria could be affected by the etiology for the development of gastric ulcer such as *Helicobacter pylori* infection and chronic use of NSAIDs. Although certain *Bacteroidetes* species could be opportunistic pathogens, the phylum *Bacteroidetes* was considered to be the most stable component in the gastrointestinal microbiota of healthy adults and act as a symbiotic microorganism in the gastrointestinal tract to play a role in protein metabolism [70]. The limitation of this study was that we did not design the groups with the administration of LBP or CPC without aspirin, and we cannot determine the effects of LBP or CPC per se on gastric biochemical indicators. Therefore, it is required further studies to verify whether LBP or CPC could have the impact and molecular regulation on gastroprotective factors, antioxidant and inflammatory markers, and gastric microbiota.

## Conclusions

In summary, after gastric ulcer caused by aspirin for 8 weeks in rats, LBP treatment for 9 weeks reduced gastric ulcer score, and CPC treatment for 9 weeks increased gastric total nitrite and nitrate levels and inhibited gastric lipid oxidation. The combination of LBP and CPC for 9 weeks elevated gastroprotective factors via increasing gastric COX-1, PGE_2_, and total nitrite and nitrate levels and enhanced antioxidative activities via inhibiting gastric lipid peroxidation. In addition, LBP and/or CPC improved gastric microbiota via increasing gastric *Bifidobacterium*. Therefore, CPC has gastroprotective effects on healing of gastric ulcer caused by aspirin, and combined LBP with CPC have better protection against gastric ulcer caused by aspirin in rats.

## Supplementary Information

**Additional file 1.** Supplementary file: **Fig. S1:** Accumulated body weight gains of rats from week 1 to week 9, **Fig. S2:** Macroscopic and microscopic observations of rat stomach tissue, **Fig. S3:** Effects of Lycium barbarum polysaccharides (LBP) and/or C-phycocyanin (CPC) on inflammatory markers in stomach tissues, **Fig. S4:** The taxa distribution of rat gastric microbiota, **Fig. S5:** Beta diversity of gastric microbiota, **Table S1:** Correlation coefficients between the relative abundance of the genus Bifidobacterium and the levels of gastroprotective factors in the stomach.

## Data Availability

The data are not publicly available. Nevertheless, the data are accessible from the authors upon reasonable request.

## Abbreviations

ANOVA: Analysis of variance
ASP: Aspirin
COX: Cyclooxygenase
CPC: C-phycocyanin
HPAEC-PAD: High pH anion exchange chromatography-pulsed amperometric detection
HRP: Horseradish peroxidase
ICAM-1: Intercellular adhesion molecule-1
IL: Interleukin
LBP: *Lycium barbarum* polysaccharides
LDA: Linear discriminant analysis
LEfSe: Linear discriminant analysis effect size
MDA: Malondialdehyde
NF-κB: Nuclear factor-κB
NGS: Next-generation sequencing
NO: Nitric oxide
NOx: Nitrite and nitrate
Nrf2: Nuclear factor erythroid 2-related factor 2
NSAIDs: Non-steroidal anti-inflammatory drugs
SOD: Superoxide dismutase
PCoA: Principal coordinate analysis
PCR: Polymerase chain reaction
PBS: Phosphate buffered saline
PGs: Prostaglandins
SEM: Standard error of means
TMB: 3,3′,5,5′-Tetramethylbenzidine
TNF-α: Tumor necrosis factor-α
UI: Ulcer index
