# Supplementary material for: Combined Lycium babarum polysaccharides and C-phycocyanin increase gastric Bifidobacterium relative abundance and protect against gastric ulcer caused by aspirin in rats
Source: Nutr Metab (Lond). 2021 Jan 6;18:4. doi: 10.1186/s12986-020-00538-9 (PMC7789774; doi:10.1186/s12986-020-00538-9)
Supplement: Supplementary file 1 — Additional file 1. Supplementary file: Fig. S1: Accumulated body weight gains of rats from week 1 to week 9, Fig. S2: Macroscopic and microscopic observations of rat stomach tissue, Fig. S3: Effects of Lycium barbarum polysaccharides (LBP) and/or C-phycocyanin (CPC) on inflammatory markers in stomach tissues, Fig. S4: The taxa distribution of rat gastric microbiota, Fig. S5: Beta diversity of gastric microbiota, Table S1: Correlation coefficients between the relative abundance of the genus Bifidobacterium and the levels of gastroprotective factors in the stomach. [file 12986_2020_538_MOESM1_ESM.pdf]

Combined *Lycium babarum* polysaccharides and C-phycocyanin increase gastric *Bifidobacterium* relative abundance and protect against gastric ulcer caused by aspirin in rats

Shu-Yu Hsieh [1], Yu Zhi Lian [1], I-Hsuan Lin [2], Yu-Chen Yang [3], Alexey A. Tinkov [4,5,6], Anatoly V. Skalny [5,6,7] and Jane C.-J. Chao [1,8,9*]

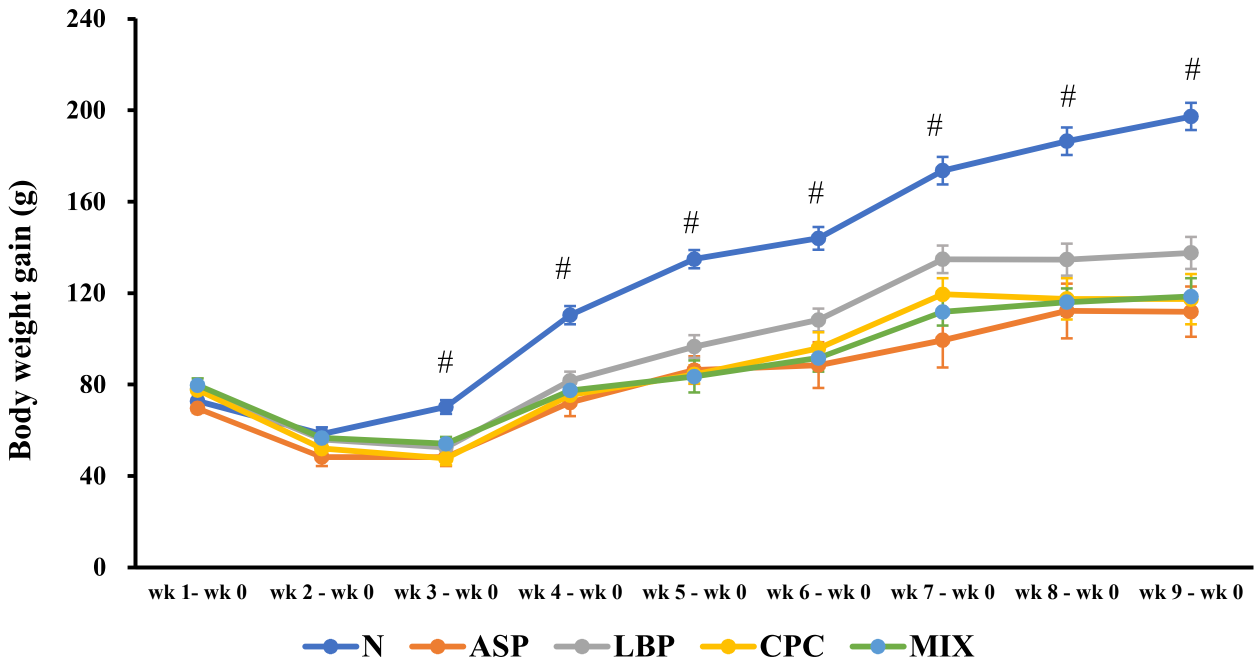

Supplementary Fig. 1 Accumulated body weight gains of rats from week 1 to week 9. N: standard powder diet; ASP: aspirin; LBP: aspirin + *Lycium barbarum* polysaccharides (LBP); CPC: aspirin + C-phycocyanin (CPC); MIX: aspirin + LBP + CPC. Data are mean ± SEM (n = 10-12 per group). Differences between the groups were determined by one-way ANOVA followed by Fisher's least significant difference test. $^{\#}p < 0.05$ compared with the ASP group.

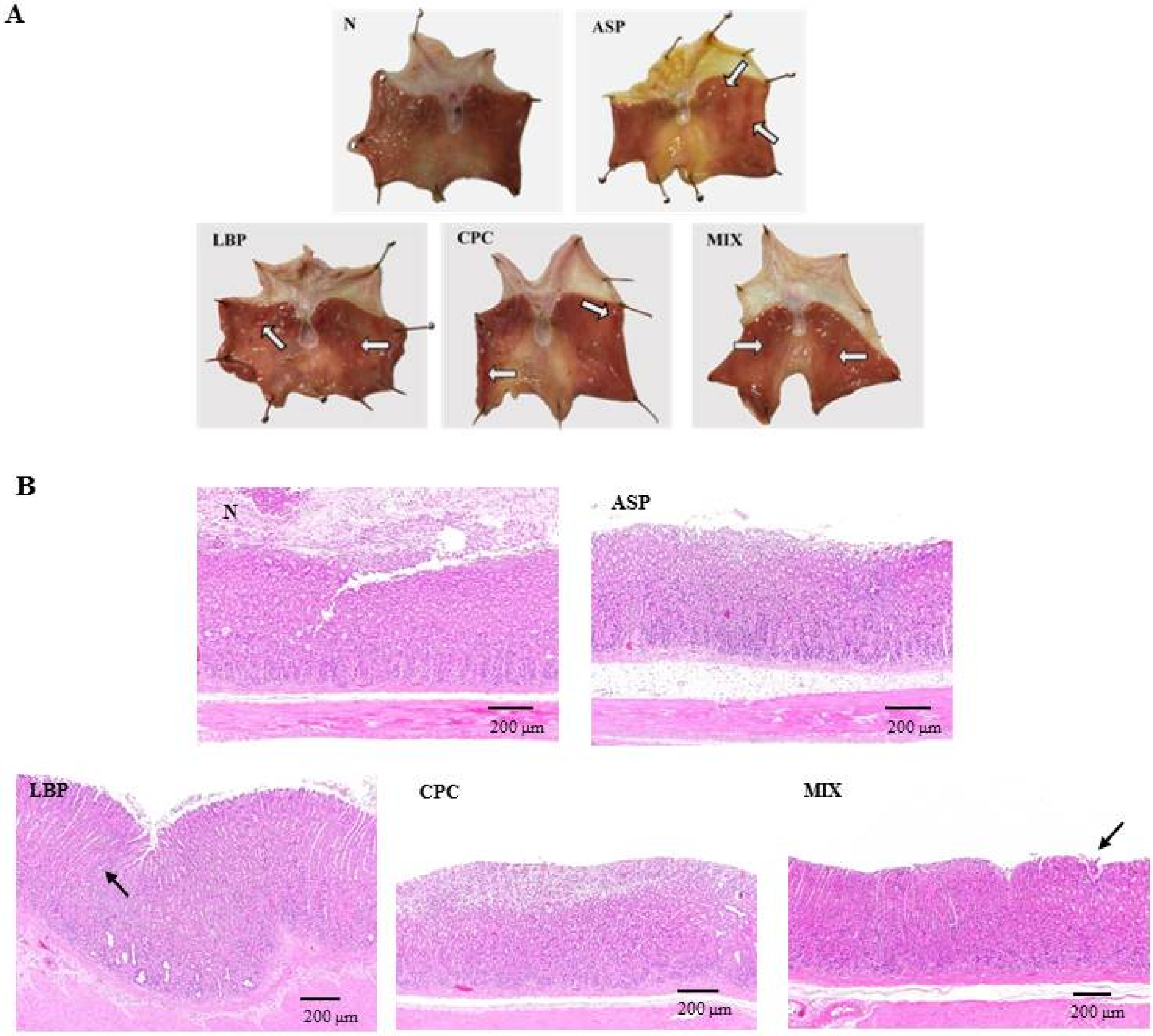

Supplementary Fig. 2 Macroscopic and microscopic observations of rat stomach tissue. Macroscopic appearance of **a** rat gastric mucosa surface and **b** histological staining by H&E (100×). N: standard powder diet; ASP: aspirin; LBP: aspirin + *Lycium barbarum* polysaccharides (LBP); CPC: aspirin + C-phycocyanin (CPC); MIX: aspirin + LBP + CPC. Hollow arrows in **a** indicate the red coloration of glandular stomach. Solid arrows in **b** indicate focal epithelial regeneration of gastric mucosa.

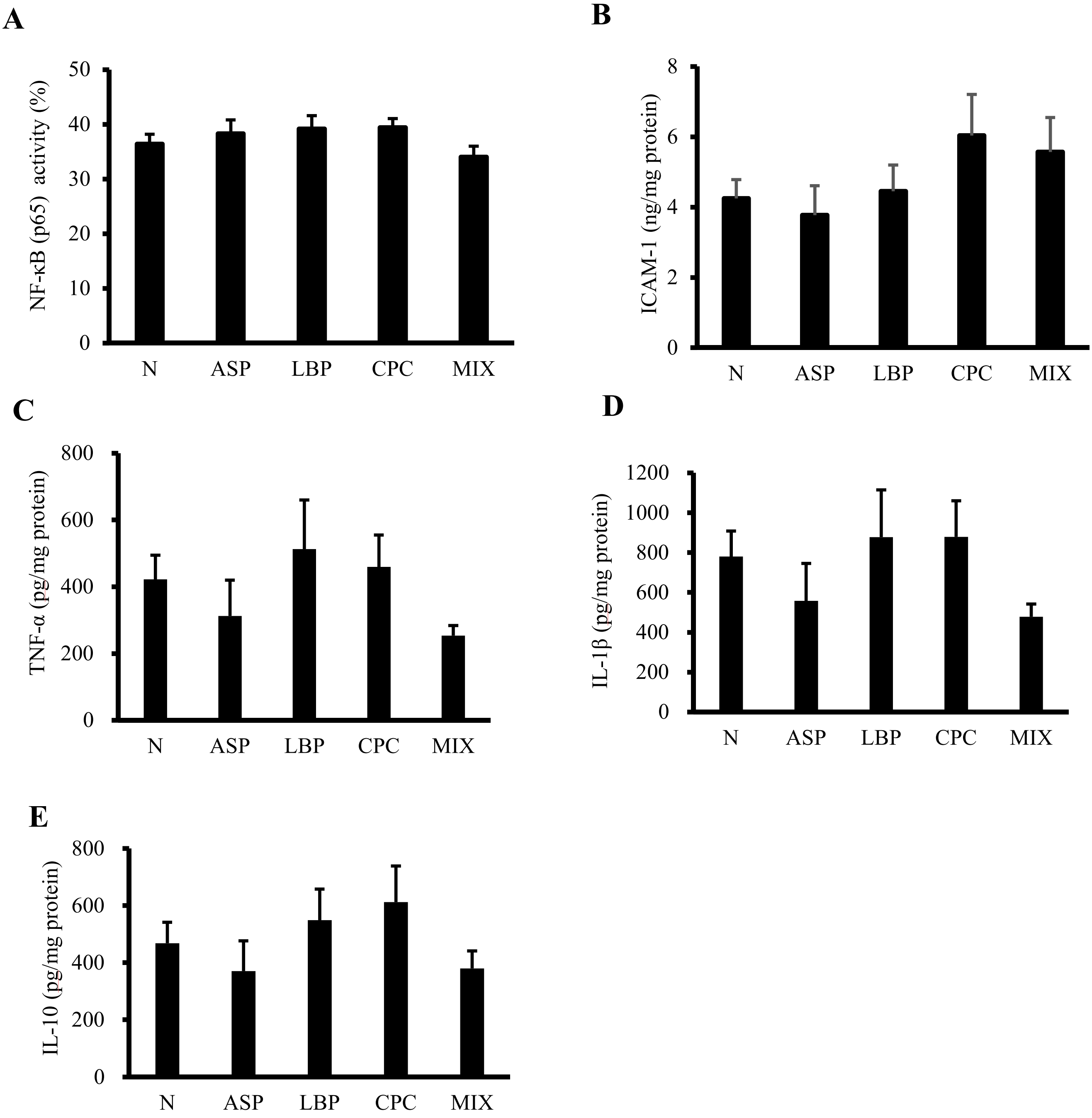

Supplementary Fig. 3 Effects of *Lycium barbarum* polysaccharides (LBP) and/or C-phycocyanin (CPC) on inflammatory markers in stomach tissues. **a** Nuclear factor-κB (NF-κB) (p65) activity, **b** intercellular adhesion molecule-1 (ICAM-1), **c** tumor necrosis factor-α (TNF-α), **d** interleukin-1β (IL-1β), and **e** interleukin-10 (IL-10). N: standard powder diet; ASP: aspirin; LBP: aspirin + *Lycium barbarum* polysaccharides (LBP); CPC: aspirin + C-phycocyanin (CPC); MIX: aspirin + LBP + CPC. Data are mean ± SEM (n = 10-12 per group). Differences between the groups were determined by one-way ANOVA followed by Fisher's least significant difference test. There were no statistical differences in all inflammatory markers between any two groups.

A

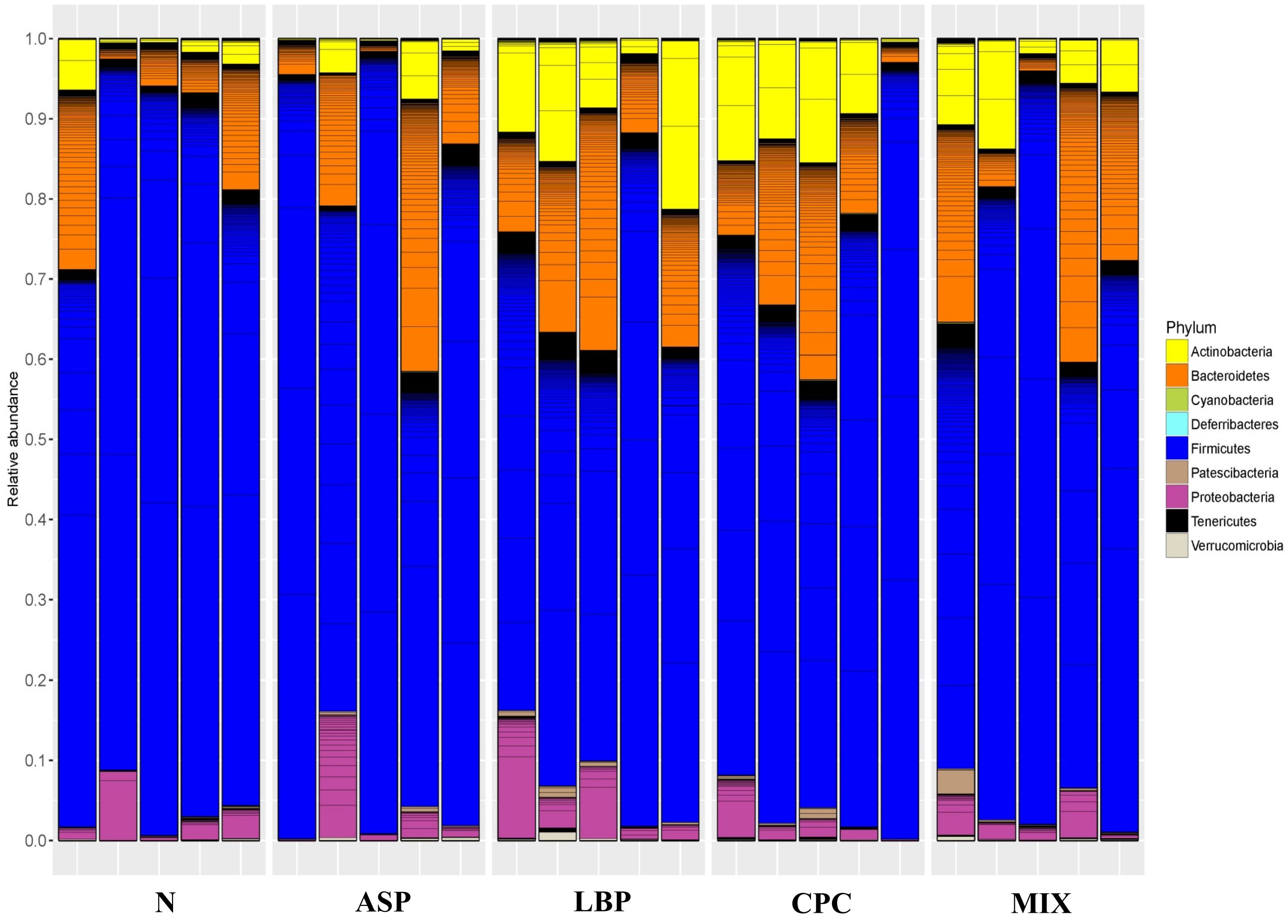
1.0
0.9
0.8
0.7
0.6
0.5
0.4
0.3
0.2
0.1
0.0
Relative abundance
N
ASP
LBP
CPC
MIX
Phylum
Actinobacteria
Bacteroidetes
Cyanobacteria
Deferribacteres
Firmicutes
Patescibacteria
Proteobacteria
Tenericutes
Verrucomicrobia

B

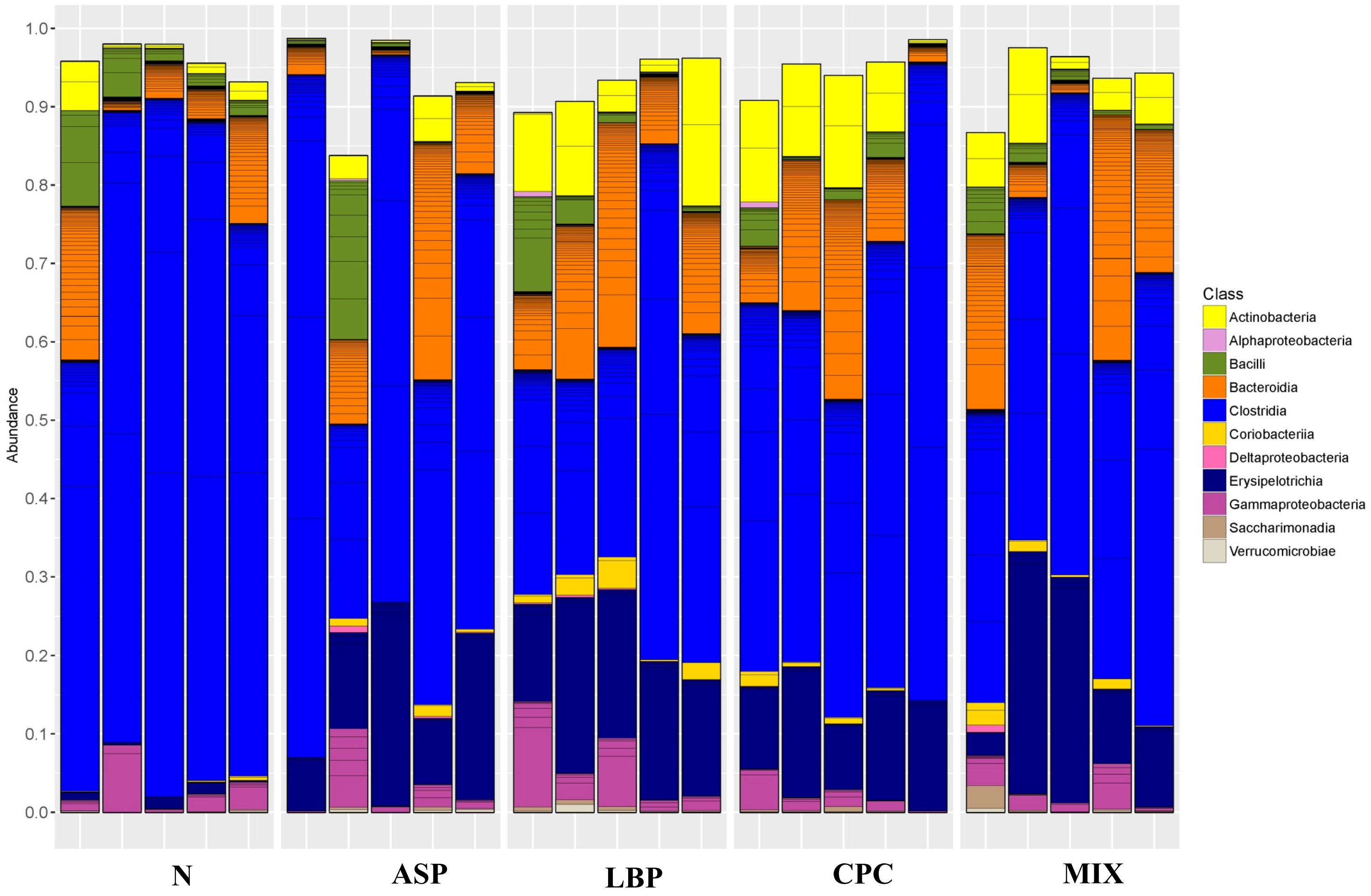
Abundance
1.0
0.9
0.8
0.7
0.6
0.5
0.4
0.3
0.2
0.1
0.0
Class
Actinobacteria
Alphaproteobacteria
Bacilli
Bacteroidia
Clostridia
Coriobacteriia
Deltaproteobacteria
Erysipelotrichia
Gammaproteobacteria
Saccharimonadia
Verrucomicrobiae
N
ASP
LBP
CPC
MIX

C

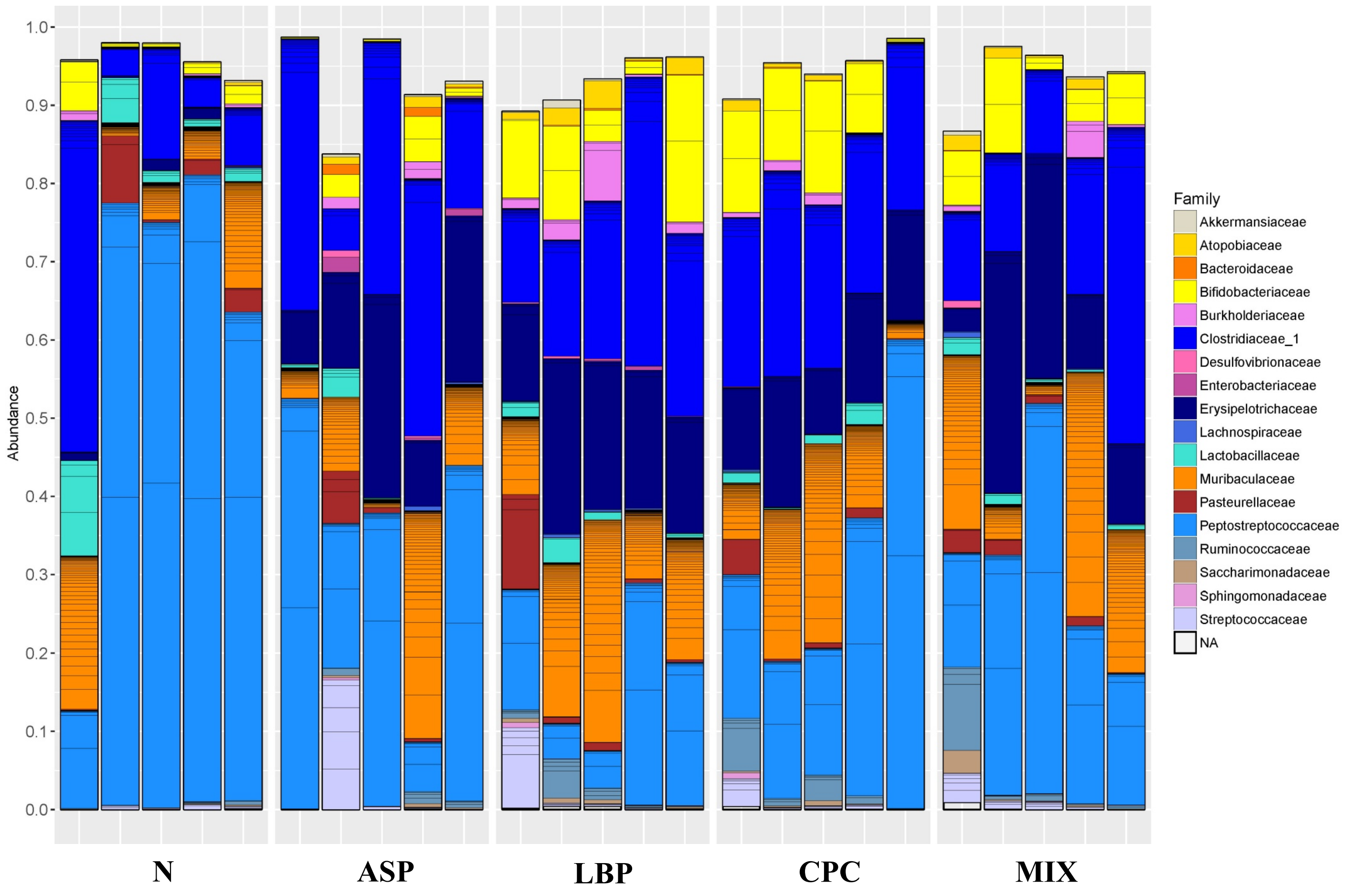
1.0
0.9
0.8
0.7
0.6
0.5
0.4
0.3
0.2
0.1
0.0
Abundance
N
ASP
LBP
CPC
MIX
Family
Akkermansiaceae
Atopobiaceae
Bacteroidaceae
Bifidobacteriaceae
Burkholderiaceae
Clostridiaceae_1
Desulfovibrionaceae
Enterobacteriaceae
Erysipelotrichaceae
Lachnospiraceae
Lactobacillaceae
Muribaculaceae
Pasteurellaceae
Peptostreptococcaceae
Ruminococcaceae
Saccharimonadaceae
Sphingomonadaceae
Streptococcaceae
NA

D

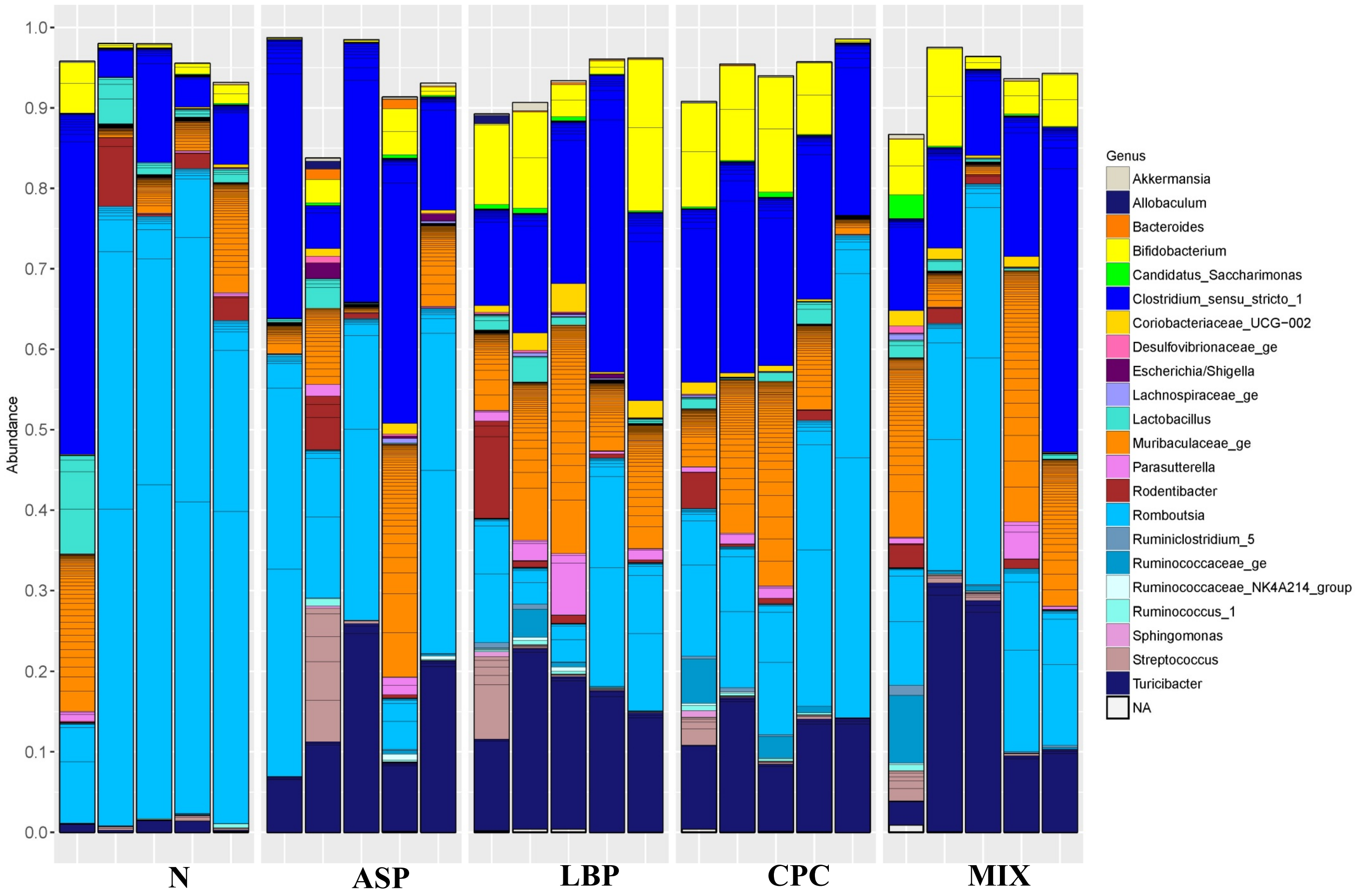

Supplementary Fig. 4 The taxa distribution of rat gastric microbiota. **a** Taxa by phylum, **b** top 100 taxa by class, **c** top 100 taxa by family, and **d** top 100 taxa by genus. N: standard powder diet; ASP: aspirin; LBP: aspirin + *Lycium barbarum* polysaccharides (LBP); CPC: aspirin + C-phycocyanin (CPC); MIX: aspirin + LBP + CPC. Each bar represents an individual sample, and 5 samples are randomly selected from each group (n = 5 per group).

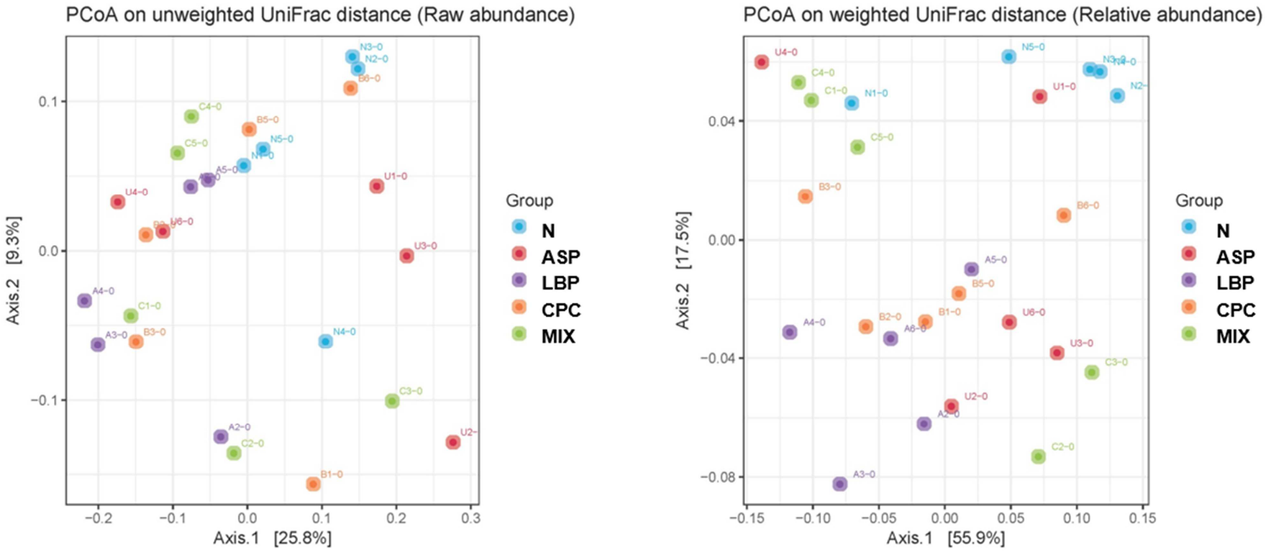

Supplementary Fig. 5 Beta diversity of gastric microbiota. N: standard powder diet (N); ASP: aspirin (U); LBP: aspirin + *Lycium barbarum* polysaccharides (LBP) (A); CPC: aspirin + C-phycocyanin (CPC) (B); MIX: aspirin + LBP + CPC (C). The left and right panels indicate raw abundance by unweighted UniFrac distance and relative abundance by weighted UniFrac distance, respectively, using principal coordinate analysis (PCoA). The 5 dots with the same color represent 5 samples from each group (n = 5 per group). There were no significant differences in beta diversity by unweighted and weighted UniFrac distances among five groups using Kruskal-Wallis one-way ANOVA.

Supplementary Table 1 Correlation coefficients between the relative abundance of the genus *Bifidobacterium* and the levels of gastroprotective factors in the stomach

	Relative abundance of *Bifidobacterium* (n = 25)	COX-1 (n = 25)	COX-2 (n = 23)	PGE_2 (n = 25)	NOx (n = 25)
Relative abundance of *Bifidobacterium*	-	0.235	0.324	-0.239	-0.083
COX-1	0.235	-	0.892^{***}	0.186	-0.083
COX-2	0.324	0.892^{***}	-	-0.082	-0.195
PGE_2	-0.239	0.186	-0.082	-	-0.092
NOx	0.085	-0.083	-0.195	-0.092	-

COX-1: cyclooxygenase-1; COX-2: cyclooxygenase-2; PGE_2: prostaglandin E_2; NOx: total nitrite and nitrate (NOx). Correlation was determined by Pearson's correlation coefficient. $^{***}p < 0.001$.
